# Transcranial photobiomodulation in children aged 2–6 years: a randomized sham-controlled clinical trial assessing safety, efficacy, and impact on autism spectrum disorder symptoms and brain electrophysiology

**DOI:** 10.3389/fneur.2024.1221193

**Published:** 2024-04-26

**Authors:** Yuliy Fradkin, Luis De Taboada, Margaret Naeser, Anita Saltmarche, William Snyder, Eugenia Steingold

**Affiliations:** ^1^Robert Wood Johnson Medical School, Rutgers University, New Brunswick, NJ, United States; ^2^JelikaLite Corp., New York, NY, United States; ^3^Chobanian and Avedisian School of Medicine, Boston University, Boston, MA, United States; ^4^Saltmarche Health and Associates, Orangeville, ON, Canada; ^5^Vyir Technology, New York, NY, United States

**Keywords:** ASD, autism, PBM, tPBM, EEG, delta waves, pediatric neurology

## Abstract

**Background:**

Small pilot studies have suggested that transcranial photobiomodulation (tPBM) could help reduce symptoms of neurological conditions, such as depression, traumatic brain injury, and autism spectrum disorder (ASD).

**Objective:**

To examine the impact of tPBM on the symptoms of ASD in children aged two to six years.

**Method:**

We conducted a randomized, sham-controlled clinical trial involving thirty children aged two to six years with a prior diagnosis of ASD. We delivered pulses of near-infrared light (40 Hz, 850 nm) noninvasively to selected brain areas twice a week for eight weeks, using an investigational medical device designed for this purpose (Cognilum^™^, JelikaLite Corp., New York, United States). We used the Childhood Autism Rating Scale (CARS, 2nd Edition) to assess and compare the ASD symptoms of participants before and after the treatment course. We collected electroencephalogram (EEG) data during each session from those participants who tolerated wearing the EEG cap.

**Results:**

The difference in the change in CARS scores between the two groups was 7.23 (95% CI 2.357 to 12.107, *p* = 0.011). Seventeen of the thirty participants completed at least two EEGs and time-dependent trends were detected. In addition, an interaction between Active versus Sham and Scaled Time was observed in delta power (Coefficient = 7.521, 95% CI -0.517 to 15.559, *p* = 0.07) and theta power (Coefficient = −8.287, 95% CI -17.199 to 0.626, p = 0.07), indicating a potential trend towards a greater reduction in delta power and an increase in theta power over time with treatment in the Active group, compared to the Sham group. Furthermore, there was a significant difference in the condition (Treatment vs. Sham) in the power of theta waves (net_theta) (Coefficient = 9.547, 95% CI 0.027 to 19.067, *p* = 0.049). No moderate or severe side effects or adverse effects were reported or observed during the trial.

**Conclusion:**

These results indicate that tPBM may be a safe and effective treatment for ASD and should be studied in more depth in larger studies.

**Clinical trial registration**: https://clinicaltrials.gov/ct2/show/NCT04660552, identifier NCT04660552.

## Introduction

Autism Spectrum Disorder (ASD) is a complex, multi-causal neurodevelopmental disorder characterized by decreased social functioning, communication impairment, and repetitive behavior (1). Many individuals with ASD have sensory abnormalities, which often lead to behavioral difficulties, such as aggression, self-injurious behavior, tantrums, irritability, and sleep disturbances. Frequently, the core symptoms of ASD and their accompanying behavioral problems interfere with the education and development of children and the well-being of their caregivers (2–4).

The etiology of ASD remains unclear, but there is evidence of a variety of morphological changes in the brain, abnormal brain cytoarchitecture, and neuroinflammation. Autistic individuals have abnormally large frontal, parietal, and temporal cortical regions during childhood, but the regions undergo premature size reductions from adolescence to late middle age, potentially due to cell death (5). They have smaller cerebellums (6–9). Cerebellar abnormalities have been associated with deficits in social cognition and repetitive and restrictive behaviors (10). They also have larger amygdalae during childhood that normalize in adulthood, enlarged hippocampi throughout all ages (11), thicker subependymal cell layers and nodular dysplasia, abnormal growth of the dentate nucleus, and dysplasia of the granule layers in the dentate gyri, all suggesting altered neurogenesis (12). The link between brain anatomy and brain function supports functional abnormalities in their pars opercularis, superior temporal cortex, middle temporal cortex, and superior frontal cortex (13). The brain cytoarchitecture of people with ASD is also different. Their brains have more neurons and fewer astrocytes in the prefrontal cortex and fewer cerebellar Purkinje and granule cells in the cerebellum (14). Of note, it has been observed that astrocytes and microglia in patients with ASD, particularly in the hippocampus and cerebellum, become reactive and release pro-inflammatory cytokines leading to chronic neuroinflammation (15).

There is also an imbalance of functional connectivity throughout the brain in individuals with ASD (16). For example, a review of twenty-nine studies on Default Mode Network (DMN) connectivity in adolescents with ASD reported a predominant lack of connectivity (17). Several resting-state functional magnetic resonance imaging (rs-fMRI) studies of the DMN in individuals with ASD have also shown it to have lower functional connectivity than the DMN in neurotypical individuals with typical development (18). These studies also showed negative correlations between the strength of functional connectivity in the resting state of DMN and traits of the autism spectrum, including social deficits in individuals with ASD.

The pathology of ASD is often associated with changes in mitochondrial function (19–24). Mitochondrial dysfunction can cause a variety of issues such as increased levels of reactive oxygen species (ROS), abnormal calcium regulation, and neurotransmitter imbalances. These problems can result in neuroinflammation, dysfunctional neuronal activity, and neuronal cell death. Singh, investigating developmental regression (DR) in children with autism, suggested that *mitochondria may represent a potential target for ASD therapeutic interventions*: “Since mitochondrial function was found to be related to ASD symptoms, mitochondria could be a potential target for new therapeutics. Furthermore, identifying individuals with mitochondrial vulnerability before DR could result in prevention of ASD” (25).

ASD is typically treated with behavioral therapies and pharmacological approaches that aim to reduce aggression (2, 26, 27). However, these approaches often have side effects, for example, sedation, anticholinergic effects, metabolic alterations, weight gain, and involuntary movements, and do not target the pathology or the main symptoms of ASD (27). Non-invasive brain stimulation may be a potentially effective approach to reduce the core symptoms of ASD (28–30).

Photobiomodulation (PBM) is a therapeutic technique that uses red or near-infrared (NIR) light from lasers or LEDs. NIR light penetrates the tissues of the scalp and skull sufficiently to deliver potentially therapeutic doses of light to neural cells in humans, doses that produced clinically significant benefits in animal models of human brain injuries and neurological disorders (31). Taking advantage of this finding, researchers have tested tPBM, red and/or NIR light applied to the scalp surface, to modulate neural cell biochemistry. Clinically, tPBM demonstrated improved neurological outcomes after acute ischemic stroke (32) and after TBI (33–36). tPBM improved language production in patients with aphasic stroke, specifically, when their DMN was stimulated (37). Additionally, several researchers have shown that tPBM attenuates the symptoms of major depressive disorder (38–42), dementia (35), and Parkinson’s disease (43). Furthermore, tPBM has been shown to improve sustained attention, short-term memory, and category learning (44–46). No side effects were reported in these studies. In addition, a mini-review of tPBM for autism (47) showed little or no evidence of side effects or toxicity on body cells.

The therapeutic effects of tPBM have been attributed to its ability to modulate cellular biochemistry (48, 49), influence brain electrophysiology (50, 51), increase cerebral blood flow (35, 37, 38, 52–54), and reduce inflammation (47). Case studies and small pilot studies have also increased functional connectivity (35, 37, 55–58). These findings suggest that exposure of neural cells to NIR light enhances their oxidative metabolism. The process is initiated by light absorption in mitochondria (59–61), specifically in cytochrome C oxidase (CCO): a large protein complex that catalyzes oxygen consumption in cellular respiration. CCO is essential for aerobic energy generation and cell survival, as cells must produce energy at a rate that matches their energy rate of consumption (62). Multiple lines of evidence suggest that CCO activity is altered in ASD, making tPBM a potential method for modulating mitochondrial function in ASD. For example, increased CCO activity in ASD has been found in muscle (20) and buccal cells (19), and in enzymology in fibroblasts, where it appears to be associated with a more normal mitochondrial morphology (63). Increased mitochondrial respiratory activity, presumably due to increased CCO activity, has been associated with the neurodevelopmental subtype of ASD (25) and is characteristic of a model of mitochondrial dysfunction in a lymphoblastoid cell line in individuals with ASD (64).

*In vitro* studies in HeLa cell cultures have shown that CCO-absorbed light promotes increased ATP synthesis and the release of mitochondrial ROS and nitric oxide (NO), both of which are critical molecules involved in multiple physiological processes and cellular pathological conditions (59, 61, 65, 66). *In vivo* studies in animal models of neurological disorders confirmed that PBM modulates mitochondrial function by increasing CCO activity in the brains of rats and mice (48, 67–69). The same researchers showed that PBM improved oxygen consumption and metabolic capacity in rat brains, leading to improved frontal cortex-based memory function. PBM reduced microglial activation and inflammation in mouse brains when NIR light was applied soon after induction of acute trauma brain injury (TBI) (70).

The cellular mechanisms by which PBM induces clinical benefits appear to be suitable for the treatment of neurodevelopmental disorders such as ASD. tPBM may be effective in reducing the symptoms of ASD due to its effects on the balance of functional brain connectivity and its anti-inflammatory properties (54).

tPBM may be effective in reducing pathological behaviors associated with ASD in a mouse model (71). Preliminary data on the use of tPBM as a therapeutic intervention for individuals with ASD are encouraging. tPBM therapy reduced repetitive and restricted behaviors in adults (72). A double-blind, randomized, sham controlled study found that tPBM therapy reduced the symptoms of ASD in children with autism (73). tPBM therapy reduced irritability in children and adolescents with ASD, a reduction that was maintained for at least 6 months (74). tPBM therapy reduced CARS and Montefiore Einstein Rigidity Scale scores (improved symptoms of ASD), and improved sleep (75).

We hypothesized that NIR light stimulation of some cortical nodes of the DMN and other areas of the brain affected by ASD (e.g., cerebellum) in young children could safely reduce symptoms, improve language, and modulate brain electrophysiology.

## Materials and methods

### Study design

This study is a randomized, double-blind, concurrent, sham-controlled trial designed to evaluate the safety and efficacy of Cognilum, an investigational medical device, for the treatment of symptoms of ASD in two- to six-year-old children.

The study protocol was approved by the institutional review board (WCG IRB approval # 1280247) and is registered at ClinicalTrials.gov (Identifier: NCT04660552).

### Sample size

The US Food and Drug Administration (FDA) is likely to make a decision regarding a minimal clinically important difference (MCID) based on a paper by Jurek et al. (76) which suggested that a clinically significant difference (CSD) is a 4.5-point reduction in CARS scores after treatment in the active group (see further discussion in the Limitations Section). We expected at least a 1- to 2-point reduction in CARS scores in the Sham group (see Results Section for the mean reductions in both groups).

This was the first human trial evaluating the Cognilum^™^ (JelikaLite Corp, New York United States) device. Without previous testing in humans to assess its safety and potential efficacy (powered to detect a CSD), we relied on published research and studies which used 11 participants (77) and 40 participants (74, 78), and estimated that a sample size of 30 participants would be adequate for statistical analysis. The sample size for this trial was set at 30 for feasibility.

## Participants

### Eligibility criteria

The study enrolled children of both sexes aged two to six years, who had previously been diagnosed with ASD by a licensed professional according to the Diagnostic Statistical Manual of Mental Disorders, Fifth Edition (DSM V). Most participants received their diagnosis at 24 months during an evaluation provided by the NYS Department of Health Early Intervention program, which typically includes a series of tests such as ADOS, CARS, TABS, and ADI-R. Approximately half of the participants in each group received one or more types of behavioral therapy (i.e., Applied Behavioral Analysis [ABA], speech therapy, occupational therapy, or physical therapy), which did not prevent them from participating in the trial, see Table 1. Exclusion criteria for the study included regular use of medications or a history of seizures. More details on the inclusion and exclusion criteria can be found in Table 2. There were no changes in the trial methods after the trial began.

**Table 1:** Demographics and baseline values of the Active vs. Sham Control groups.

	Active group *N* = 16	Sham group *N* = 14
*Age, years, mean (SD)*	4.9 (1.25)	4.6 (1.16)
*Sex, n (%)*		
Male	14 (87.5)	10 (71.4)
Female	2 (12.5)	4 (28.6)
*Ethnicity, n (%)*		
White	11 (68.75)	11 (78.57)
Asian/South Asian	2 (12.5)	3 (21.43)
Black	2 (12.5)	0
Hispanic	1 (6.25)	0
*Verbal status n (%)*		
Verbal	7 (43.75)	11 (78.57)
Non-verbal	9 (56.25)	3 (21.43)
*Receiving therapy n (%)*		
Yes	12 (75%)	11 (78.57%)
No	4 (25%)	3 (21.43%)
*Baseline CARS score, mean (SD)*	43.5 (5.7)	40.6 (7.2)

**Table 2:** Inclusion and exclusion criteria.

*Inclusion criteria*
1.Male or female participants between 2 and 6 years of age (inclusive)
2.Previously diagnosed with moderate or severe ASD by a licensed professional
3.Participants may be receiving any behavioral intervention therapy (e.g., ABA) during the course of the treatment
4.Parents of participants must understand the nature of the study
*Exclusion criteria*
1.Participant child is experiencing severe self-injurious behavior or severe aggressive behavior to self or others (within the past 7 days)
2.Participant has been diagnosed with another psychiatric or neurological disorder (e.g., epilepsy) or has a history of seizures or have exhibited symptoms of major psychiatric disorders within the last 30 days
3.Participant has an unstable medical condition that requires clinical attention
4.Participant has a significant skin condition at the procedure sites
5.Participant has an implant of any kind in the head
6.Participant has receiving medication on a regular basis
7.Any use of light-activated drugs
8.Participant is a member of investigators’ immediate family

### Randomization

Participants were randomly assigned to the Active or Sham Control groups using a random sequence generation program at www.random.org. The random sequence was created beforehand, and assignment to each group was conducted by a research assistant who was not involved in the evaluation or enrollment of the participants. The enrollment was conducted by licensed clinicians, the first and last authors in the study. No restrictions were applied to the randomization process. Before enrollment, each participant signed an informed consent form acknowledging that they had the same chance of being assigned to the Active or Sham condition.

## Intervention

### Setting and locations

The study was conducted in two private medical offices (one location was in Brooklyn NY and another one was in Manhattan, NY). Both locations were approved by the Institutional Review Board. Both locations are easily accessible by public transportation (subway and bus) and by car (with garages available nearby). New York City has a diverse population, both racially and economically, with autism prevalence rates similar to those of the rest of the country, approximately 3% (Autism and Developmental Disabilities Monitoring Network, 2018).

### Treatment device

Treatment with tPBM was administered using Cognilum^™^, an investigational medical device specifically designed to treat ASD in young children. The manufacturer is Sterling Medical Devices, Moonachie, NJ 07074. Upon reviewing JelikaLite LLC’s application for risk review classification, on October 14, 2020, the FDA concluded that the proposed clinical investigation involving Cognilum and autistic children aged two to six was a nonsignificant risk (NSR) device study. This classification was received because the Cognilum device does not fulfill the criteria for a significant risk (SR) device as defined under 21 CFR 812.3(m) of the investigational device exemptions (IDE) regulation (21 CFR 812). Subsequently, on December 29, 2021, based on the results of this study, the FDA awarded Cognilum a “Breakthrough Device Designation.”

**Figure 1:**
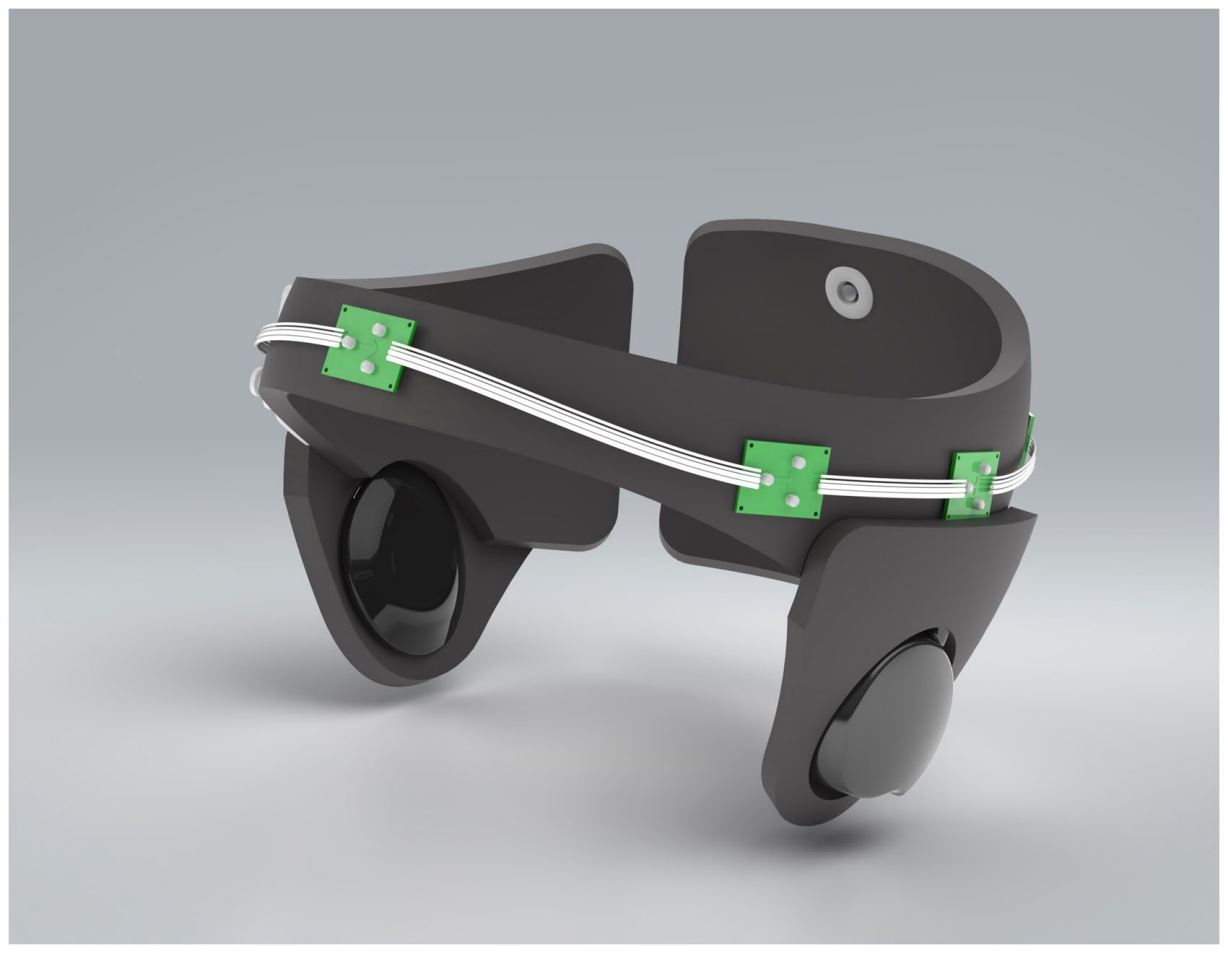
Image of the Cognilum device providing tPBM treatment.

The device weighed approximately 500 grams, was wireless, comfortable for children to wear due to the soft materials used in its construction and did not impede children’s mobility during treatment. The device was adjustable using Velcro to fit children aged two to six years. The current version of the Cognilum device cannot be used with any other device simultaneously (i.e., EEG or pacemakers). The battery used was rechargeable and the maximum use of the battery was several hours. The device specifications included 6 LEDs emitting pulsed light (40 Hz) at a wavelength of 850 nm, with a total maximum power less than 300 mW. The internal design of the device included a control panel, wires, and LEDs, as shown in Figure 1.

### Dosing and blinding

All LEDs in the investigational device could simultaneously deliver pulses of NIR light at 40 Hz, 300 mW maximum power to the patients’ scalps, hypothetically modulating mitochondrial function and inducing functional brain connectivity, across several selected brain areas. Treatment sessions were administered twice a week for 8 weeks. To avoid inducing hyperactive behavior, the duration of treatment gradually increased over the course of each subsequent session, up to 6 minutes, and then it was maintained constant.

For the Sham condition, the device performed identically to the active device in all observable behaviors, with the exception that it did not emit any NIR light. The research assistants set the device as active or sham, based on the randomization sequence that was available to them. The experience of the participants during both active and simulated conditions was indistinguishable. The NIR wavelength used by Cognilum (850 nm) is not visible to the human eye and did not cause tissue heating at the administered dose. Caregivers, participants, and assessors were blinded to whether light stimulation was administered.

### Treatment procedure

The principal investigator who is the first author (a board-certified pediatric psychiatrist) and the last author, a licensed clinical psychologist who frequently diagnoses children using CARS, conducted the initial evaluation in person at the evaluation site. The evaluation included administering initial and final CARS. The initial CARS evaluation was performed immediately before the first treatment session and lasted about an hour. The treatment session was administered twice a week for 8 weeks. Each treatment session lasted approximately 30 minutes. Each treatment session involved collecting EEG data for approximately 10 minutes, depending on the tolerance of each child, tPBM treatment for five to six minutes, followed by a second EEG data collection for another 10 minutes, in as many as participants possible. During tPBM treatment sessions, participants were allowed to walk around the office, watch cartoons or play with toys. Participants were encouraged to sit quietly during EEG collection, sitting on their parents’ lap and held by their parents to minimize movement.

## Outcomes

### Primary outcomes

Childhood Autism Rating Scales, Second Edition (CARS, 2^nd^ Edition), were evaluated before (baseline) and after the course of treatment. CARS is a validated clinical rating scale that can be used by a trained clinician to rate items indicative of ASD after direct observation of the child (79). European Medicines Agency (EMA) guidelines recommend it as an outcome measure for the clinical development of treatments for ASD. Furthermore, a consensus study has been conducted on the minimal clinically important difference in core symptoms resulting from therapeutic interventions (76). The scale consists of fifteen items that correspond to the different core domains (e.g., verbal communication, emotional response, and relationships with people) that can be affected by ASD. Total scores can range from a low of 15 to a high of 60; scores below 30 indicate that the individual is in the nonautistic range, scores between 30 and 36.5 indicate mild to moderate autism, and scores from 37 to 60 indicate severe autism (76).

### Secondary outcomes

Weekly interviews were conducted with each parent about changes in child behavior and functioning by a blinded research assistant by phone weekly. Parents were asked to maintain a diary documenting their child’s behavior, including newly produced words, changes in comprehension of instructions, consistency of eye contact, sleep patterns, tantrum severity and frequency, anxiety severity, social interaction, and eating behavior. Furthermore, qualitative data was collected through brief weekly interviews with blinded research assistants who were unaware of the experimental condition of the participant. These qualitative data provided further information.

The parents were asked the following questions:

- How many tantrums/meltdowns did your child have this week?
- On average, how many times did your child wake up at night this week?
- Did your child start to produce new words? Which/how many?
- Did you notice any changes in your child’s command following and overall responsiveness?
- Did you notice any changes in your child’s level of anxiety (e.g., any changes in his/her fear of other people).
- Did you see any changes in your child’s ability to establish and maintain eye contact?
- Did you see any changes in your child’s eating and chewing?
- Is there anything else you would like to share about changes in your child’s behavior with us? Anything that you feel is important or could potentially be important for the study.

- This last open question was specifically asked to grasp any unexpected and unforeseeable side effects or benefits from treatment.

Although tPBM technology is not known to cause any hair or skin disorders, and the same applies to EEG caps, the last open question would have grasped these data.

### Exploratory outcomes

EEG signals were collected in children before and after each treatment session. Data collection was performed using a Brain Scientific-produced clinical grade device, called NeuroEEG. The device was a thirty-two-channel dry electrode pediatric EEG softcap. The company has since started to manufacture disposable EEG caps, and this particular device has since been discontinued because it is reusable. The EEG was collected only for those children who tolerated wearing the EEG cap and could sit quietly for a period of time.

## Data collection and statistical analysis

### Primary analyses

The last author, a licensed psychologist experienced in the use of CARS in clinical practice, conducted blinded Before and After CARS ratings for all participants. The final CARS evaluations were performed immediately after the final treatment session. CARS scores were analyzed using the independent sample *t*-test. We compared the differences from the baseline between both groups, calculating both the average difference and 95% CI within each group and between groups.

### Secondary outcomes

Responses to the eight weekly questions were scored as either: a positive change representing an improvement with a value of +1, a negative change representing a regression with a value of −1, or a third option without noticeable change with a value of 0. Any appearance of a side effect (e.g., hyperactivity, transient tics) was given a score of 1 if the subject presented with that symptom or a score of 0 if that symptom was not reported. Each subject received two final cumulative scores, representing behavioral changes addressed by the above questionnaire and the appearance of side effects. The scoring of the behavioral data was performed by an analyst (fifth author of the paper) who was fully blinded to the experimental hypothesis and the treatment conditions of the participants. Cumulative scores were compared using a Wilcoxon–Mann–Whitney two-sample rank sum test.

### Exploratory analyses

The analytical team, fully blinded to both the experimental hypothesis and the condition of the participants, removed artifacts due to the movement of the participants manually. The frequency power was measured for each participant during the trial time (for those who tolerated wearing the EEG cap). The average power of each wavelength (alpha, beta, theta, and delta) was analyzed. Data analysis was performed using SciPy^1^ and Statsmodels.^2^ Matplotlib^3^ was used to plot the data.

The study employed Mixed Linear Models to analyze the effects of a treatment versus sham on two dependent variables, Net_theta (the average power of theta waves and Net_delta) (the average power of delta waves), across both physical time and a scaled time period.

The scaling of data across was applied to all data, uniformly, for all waves, and it is unlikely that the reported results are achieved solely due to scaling. Net_delta and Net_theta were used as measures, which lead to 48 total number of observations.

After conducting the statistical analysis over actual time, the following scaling was performed: the time scale was standardized so that the first observation for each participant was at point 0 and the last observation was at point 1. The time scaling accounted for individual timelines, with 0 representing a patient’s first day and 1 their last day in the study: Time_scaled_ = (Time – Time_start_) / (Time_end_ – Time_start_).

Additionally, the power of each brain wave (alpha, beta, theta, and delta), measured in percentages of 100, was analyzed over time. EEG power represented the amount of activity in certain frequency bands of the signal (80).

### Exploratory post-hoc analysis

We used linear correlation (Pearson’s R) to test the relationship between the power of theta and delta waves. We also used linear correlation (Pearson’s R) to test the relationship between changes in the power of theta and delta waves and changes in the CARS scores of participants.

### Interim analysis

There were no predetermined stopping guidelines for the interim analysis of data. In case of adverse effects, the Institutional Review Board would have been notified and, if necessary, the study stopped. There were no changes in the trial results after the start of the study.

## Results

### Participants

Thirty participants completed this trial, according to the original Institutional Review Board approval; 16 participants were in the Active group.

### Recruitment

Participants were recruited through social networks, local schools, and centers that provide behavioral therapy to children diagnosed with ASD. “Trialfacts”^4^ assisted with the recruitment. The study began in March 2021. The study ended in October 2021. The recruitment period was approximately 6 months. The trial was completed once the Institutional Review Board-approved 30 participants completed the entire course of the trial. The day the first participant started was 17 March 2021 and the day the last participant completed the study was 28 October 2021.

### Study flow

**Figure 2:**
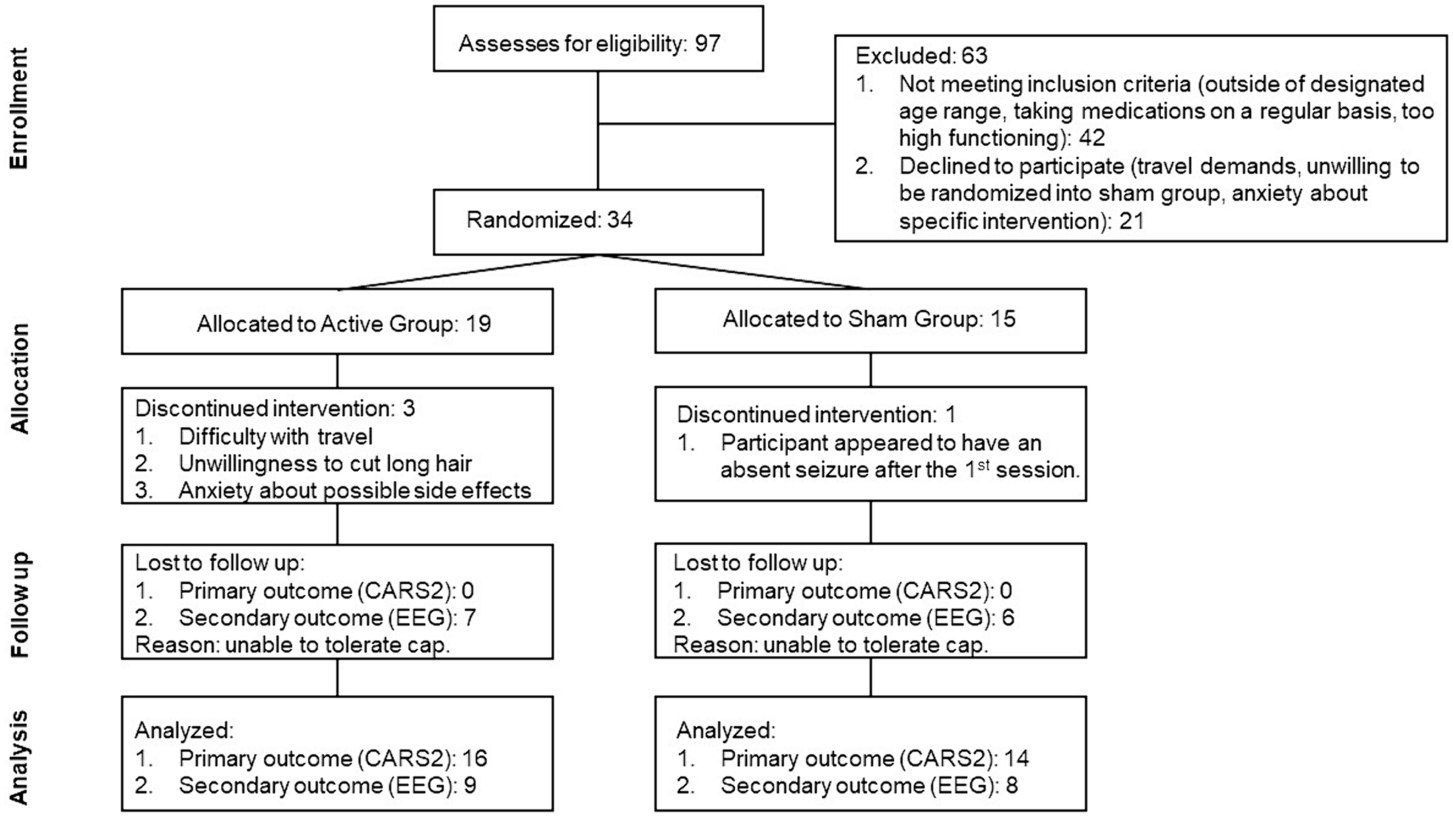
Flow diagram representing the screening and enrollment of the participants as well as the number of participants whose data was analyzed.

The study initially recruited 34 participants, but four were lost and replaced throughout the trial. A participant withdrew immediately after the first session, as it appeared that he had an absence seizure. A seizure was never confirmed; this child was randomized to a sham condition. The parents of another participant decided not to continue after one session. One participant was replaced after one session due to their parents’ refusal to cut the child’s long hair. Another participant was replaced after the first session because his mother found it difficult to travel to the experimental site. Figure 2 shows the flow chart of participant selection and recruitment, as well as their attrition during the trial.

Initially, there were 15 participants randomized to the Active and Sham groups. However, due to an experimental error, treatment was mistakenly administered to a replacement participant who was randomized to the Sham group, in the final analysis there were 16 children in the Active group and 14 children in the Sham group.

### Treatment experience

The experience of the participants under the Sham and Active conditions was identical: The device was placed on their heads and activated by the research assistant. Children were allowed to watch cartoons or YouTube videos or play with their parents or research assistants. EEG was collected from participants in both Active and Sham groups.

## Outcomes

### Baseline data

At the beginning of the trial, an initial evaluation was performed to assess the severity of ASD in participants using the CARS score. Table 1 displays the mean, median, and range of the participant scores. Most participants had moderate to severe ASD, with a CARS score higher than 35. There were no significant differences in age, gender, ethnicity, language status, treatment status, or CARS scores between the Active and Sham groups.

### Primary outcome

Every participant who completed the trial was evaluated with CARS post-treatment. There were no missing CARS posttreatment evaluations (see Figure 2).

In the Active group, the before treatment CARS scores had a mean score value of 43.5 (*n* = 16) with a standard deviation (SD) of 5.7, the after-treatment scores had a mean score of 33.7 with an SD of 5.0. In the Sham group, the before treatment CARS scores had a mean score value of 40.6 (*n* = 14) with SD of 7.2, the after-treatment scores had a mean score of 38.0 with SD of 8.4. Table 3 shows the mean (M) and SD of the CARS scores for the Active and Sham groups before and after treatment. The difference in the change in CARS between the two groups was 7.23 (95% CI 2.357 to 12.107, *p* = 0.01).

**Table 3:** CARS scores before and after treatment statistics for Active and Sham groups.

	Active group	Sham group	Difference between groups
No. of patients	16	14	
Mean (SD)			
Before	43.5 (5.7)	40.6 (7.2)	
After	33.7 (5.0)	38.0 (8.4)	
Mean change (95% CI)	9.875 (7.541–12.109)	2.643 (1.973–7.258)	7.23 (CI: 2.357–12.107) (*p* = 0.01)

In addition to analyzing the changes in CARS scores based on averages, we also conducted respondents-based analyses, quantifying the number of participants in Active and Sham groups, whose change in CARS was greater than 4.5 points (as it has been suggested by Jurek et al. (76) to be the minimal clinically important difference). The result was significant X^2^ = 8.48, *p* = 0.004. By the end of the trial, 87% of the participants in the Active group (14) had achieved an equal or greater than 4.5 point reduction in CARS scores, only 35% of the participants of Sham group (5) achieved an equal or greater than 4.5 point reduction in CARS scores. The number of subjects that achieved this minimal clinically important difference in the Active group was statistically significantly higher compared to the Sham group. Therefore, the treatment was considered to have clinically meaningful efficacy.

## Secondary outcomes

### Positive effects

Statistical analysis indicates a significant difference between parental ratings of the above eight categories (Secondary Outcomes) of behavior between the Active and Sham groups. The median cumulative changes in behavior [quartile] of the Active and Sham groups, respectively, were 20 [19, 21] and 16 [15.1, 16.9] (Wilcoxon rank sum W = 163.5, nA = 16 nS = 14, *p* = 0.05 two-tailed, rpb = 0.41).

### Side effects

During the trial, some participants experienced side effects, which were expected based on previous studies on tPBM (40, 81), and had been reported to the Institutional Review Board as a possible side effect before the study. A potential reason for these side effects is the increase in cerebral blood flow because of tPBM treatment. There were no serious adverse events during the study.

Four participants in the Active group exhibited overexcitement during the first 3 weeks of the trial, as noted by their therapists and teachers, who were unaware of their participation in the study. Overexcitement included running around excessively and pounding on the chest in one participant. Two children in the Active group also reported mild headaches, which resolved after several sessions and could have been due to increased cerebral blood flow. A mother gave her child Tylenol to alleviate the discomfort, but the headaches resolved on their own by session 6. One participant in the active condition experienced an increase in transient tics, which were present prior to the study and resolved after completion of the trial. Consistent with previous studies, several participants exhibited increased irritability; however, this research did not measure irritability on a separate scale. CARS score includes dimensions related to irritability (i.e., emotional response). Despite these side effects, all parents of the affected participants chose to continue with the trial as they observed visible improvements in their children’s eye-contact, concentration, and receptive and expressive language.

None of the participants with active or sham conditions reported experiencing skin or hair disorders during or after the trial. The difference in the appearance of side effects between the Active and Sham groups was not significantly different. The median (quartile) of the Active and Sham groups, respectively, were 2.5 [2, 4] and 1.5 [1, 3.8] (Wilcoxon rank-sum W = 207, nA = 16, nS = 14, *p* = 0.69 two-tailed, rpb = 0.08). All side effects were mild and resolved quickly (Table 4).

**Table 4:** Parental interviews: side effects.

Grade	Active group *N* = 16		Sham group *N* = 14	
	*n* (% of patients)	*n* (% of events)	*n* (% of patients)	*n* (% of events)
Adverse events after allocation				
Mild	0	0	0	0
Moderate	0	0	0	0
Severe	0	0	0	0
Side events after allocation	0	0	0	0
Mild	14 (87.5%)	2.5 [2, 4] 100%	10 (71.4%)	1.5 [1, 3.8] 100%
Moderate	0	0	0	0
Severe	0	0	0	0
Total	14	2.5	10	1.5
Serious adverse events after allocation				
Total	0	0	0	0

### Exploratory outcomes

Seventeen out of thirty participants tolerated wearing an EEG cap in at least two sessions, and two observations were lost due to experimental error. After data cleaning (removal of motion artifacts), forty-eight observations were available. A mixed linear model was used to analyze changes in delta and theta over scaled time.

We observed trends in delta and theta bandwidths and used Mixed Linear Models to evaluate the effect of a treatment versus sham on two health markers, Net_theta and Net_delta across time. If there were two observations per participant within 1 day, the average power of these two observations was taken. The model includes fixed effects for treatment, scaled time, and their interaction, as well as random effects to capture patient-specific variability. The analysis aims to provide information on both the overall efficacy of the treatment and its interaction with time, considering the differences between individual patients.

We noticed a decrease in the power of delta waves over time in the active group, and the increase in the power of theta waves over time in the active group, prompting us to examine the correlation between CARS and EEG. Our objective was to explore the association between behavioral data (measured by CARS scores) and EEG (measured by delta and theta power over time). This is an exploratory study, no corrections were made for multiple comparisons, and crude *p*-values were reported. Tables 5, 6 present the EEG analysis of Net_delta values and Net_theta using mixed linear models through physical time.

**Table 5:** Mixed linear models for power of Delta waves by unscaled time.

	Coef.	SE	*p* value	[95% CI]	
Intercept	25.89	2.608	0	20.779	31.001
Group (Active vs. Sham)	−1.841	3.806	0.63	−9.3	5.619
Time	−0.141	0.06	0.02	−0.258	−0.023
Interaction group with time	0.144	0.101	0.16	−0.055	0.342
Group var	42.608	4.522			

**Table 6:** Mixed linear models for power of Theta waves by unscaled time.

	Coef.	SE	*p* value	[95% CI]	
Intercept	54.764	3.064	0	48.758	60.769
Group (Active vs. Sham)	6.357	4.466	0.16	−2.395	15.11
Time	0.061	0.07	0.38	−0.076	0.199
Interaction group with time	−0.1	0.118	0.4	−0.332	0.132
Group var	58.517	5.73			

While changes over time were observed in Net_delta (Coefficient = −0.141, 95% CI -0.258 to −0.023, *p* = 0.02), no trend in differences between the Active group and the Sham group could be identified.

To ensure that data from all participants had equal weight in the analysis, the time scale was standardized so that the first observation for each participant was at point 0 and the last observation was at point 1. The time scaling accounted for individual timelines, with 0 representing a patient’s first day and 1 their last day in the study: Time_scaled_ = (Time – Time_start_) / (Time_end_ – Time_start_).

Table 7 presents the EEG analysis of Net_delta values *across scaled* time using mixed linear models.

**Table 7:** Mixed linear models for power of Delta waves by scaled time.

	Coef.	SE	*p* value	[95% CI]	
Intercept	27.936	2.994	0	22.07	33.8
Group (Active vs. Sham)	−4.261	4.225	0.313	−12.5	4.02
Scaled_Time	−6.713	2.963	0.023	−12.5	0.907
Interaction group with Scaled_Time	7.521	4.101	0.067	−0.52	15.56
Group var	43.453	4.613			

**Figure 3:**
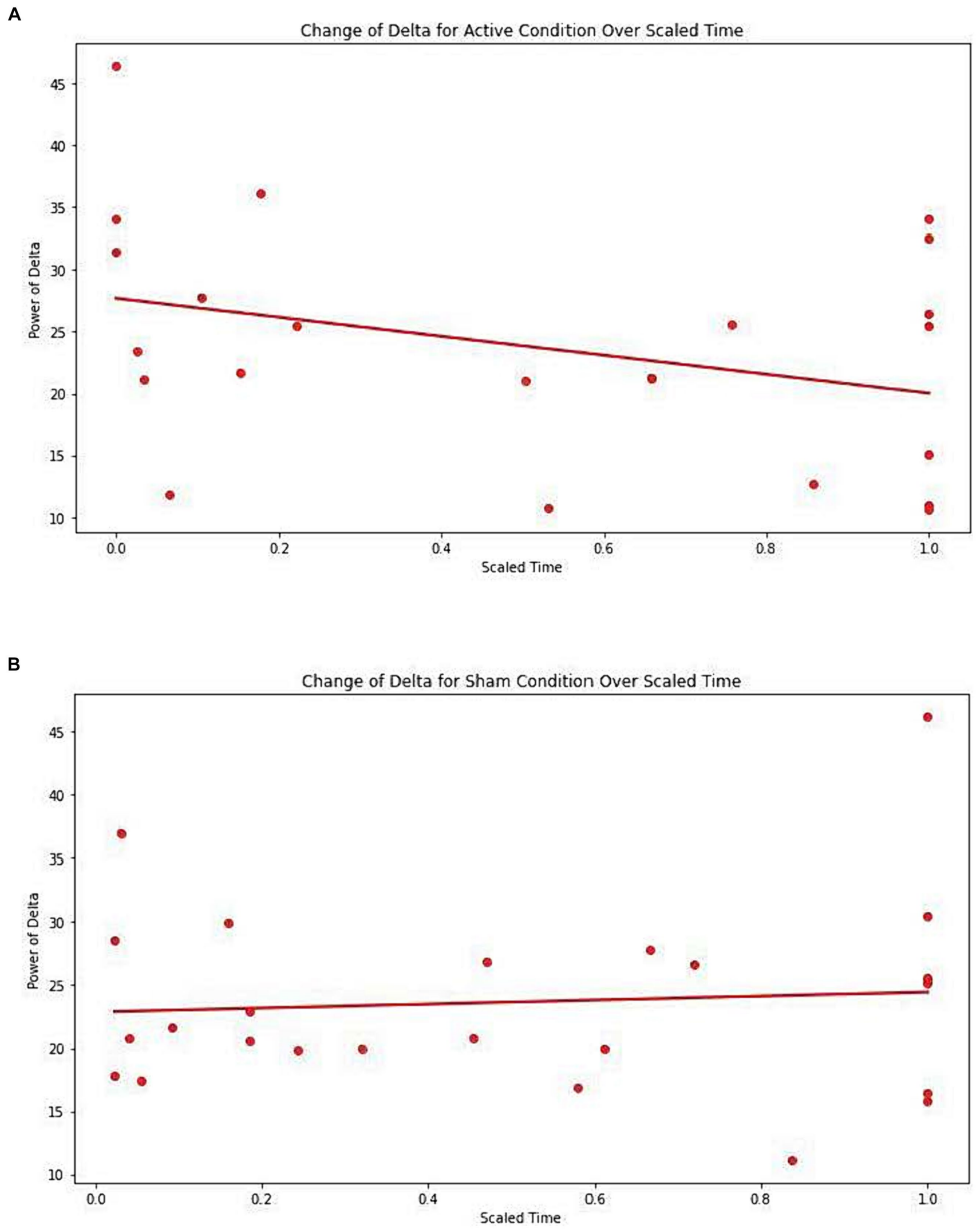
**(A)** Change of Delta for active condition over scaled time. Each dot represents each individual observation. The line is the regression line. X axis: scaled time. Y axis: power of delta. **(B)** Change of Delta for sham condition over scaled time. Each dot represents each individual observation. The line is the regression line. X axis: scaled time. Y axis: power of delta.

No significant difference was observed between conditions (Active vs. Sham) for Net_delta (Coefficient = −4.261, 95% CI -12.542 to 4.020, *p* = 0.313), while we observed a significant effect with Scaled Time (Coefficient = −6.713, 95% CI -12.520 to −0.907, *p* = 0.023). An interaction between Active vs. Sham and Scaled Time was noted (Coefficient = 7.521, 95% CI -0.517 to 15.559, *p* = 0.067), suggesting a potentially greater decrease in delta power in the Active group over time compared to the Sham group (Figures 3A,B).

Table 8 presents the EEG analysis of Net_theta values across scaled time using mixed linear models.

**Table 8:** Mixed linear models for power of Theta waves by scaled time.

	Coef.	SE	*p* value	[95% CI]	
Intercept	52.044	3.437	0	45.31	58.78
Group (Active vs. Sham)	9.547	4.857	0.049	0.027	19.07
Scaled_Time	6.429	3.29	0.051	−0.02	12.88
Interaction group with Scaled_Time	−8.287	4.547	0.068	−17.2	0.626
Group var	60.07	6.001			

**Figure 4:**
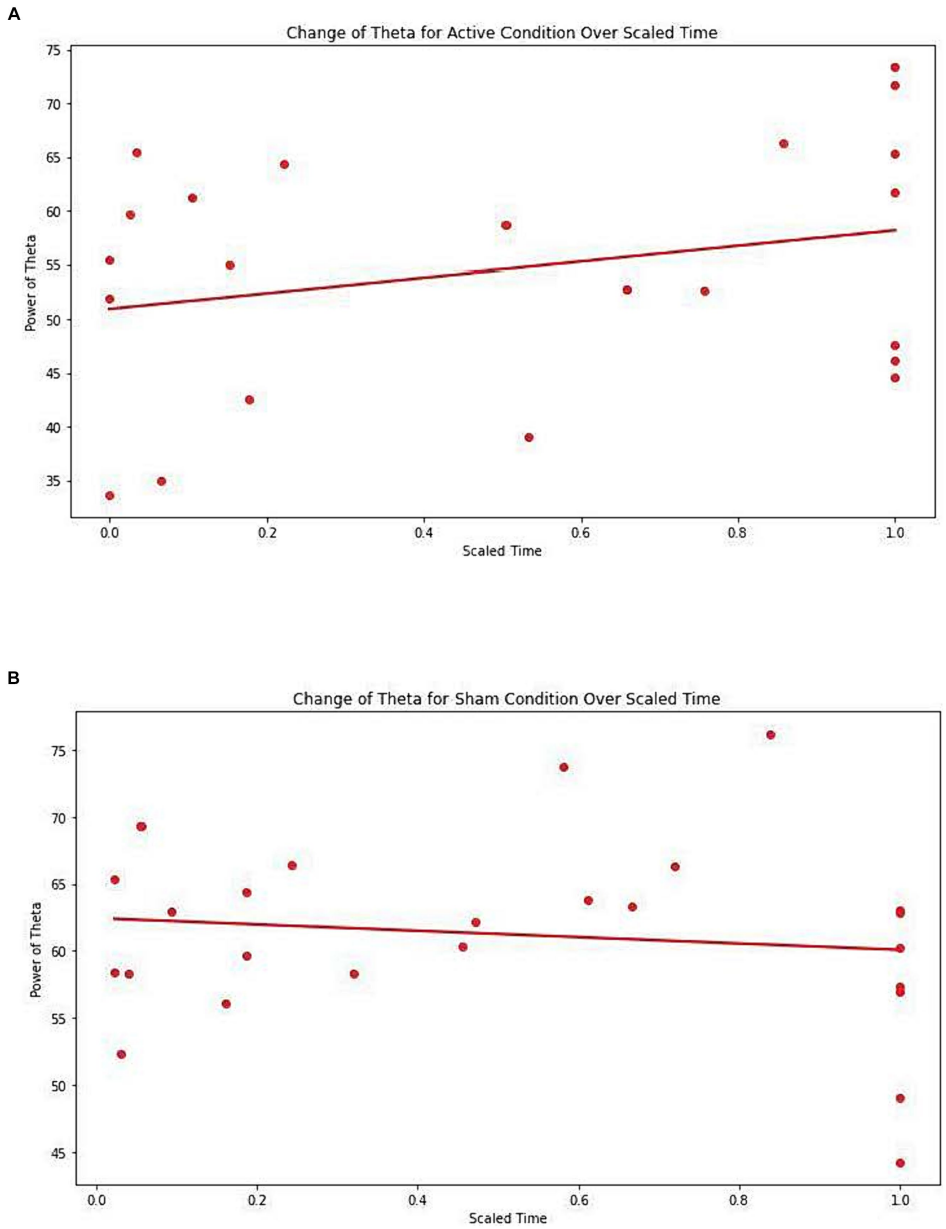
**(A)** Change of Theta for active condition over scaled time. Each dot represents each individual observation. The line is the regression line. X axis: scaled time. Y axis: power of Theta. **(B)** Change of Theta for sham condition over scaled time. Each dot represents each individual observation. The line is the regression line. X axis: scaled time. Y axis: power of Theta.

There was a significant difference observed between conditions (Active vs. Sham) for Net_theta (Coefficient = 9.547, 95% CI 0.027 to 19.067, *p* = 0.049), and a significant effect with Scaled Time (Coefficient = 6.429, 95% CI -0.020 to 12.877, *p* = 0.051). A marginal interaction between Active vs. Sham and Scaled Time was noted (Coefficient = −8.287, 95% CI –17.199 to 0.26, *p* = 0.068), suggesting a potentially greater increase in theta power in the Active group over time compared to the Sham group (Figures 4A,B).

### Exploratory post-hoc analysis

**Figure 5:**
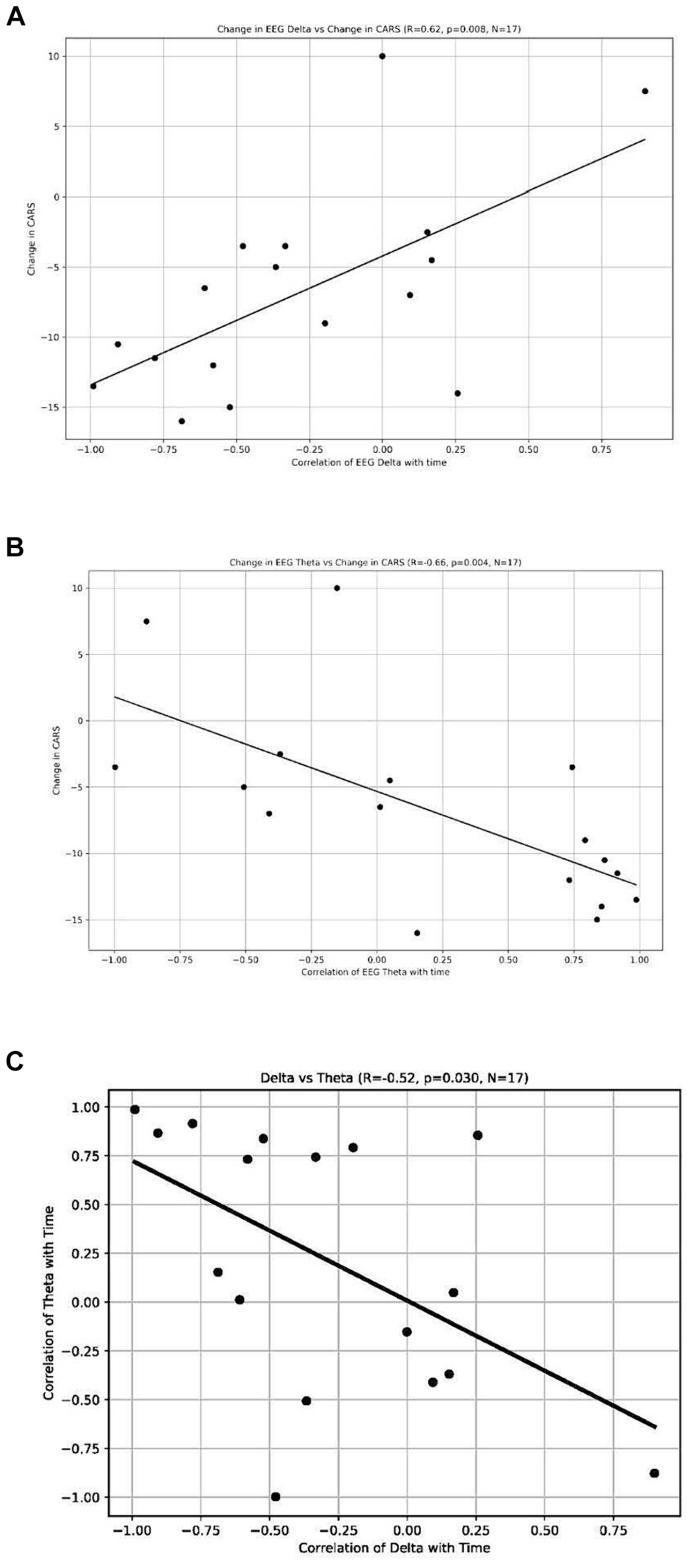
**(A)** Change of EEG Delta vs. Change in CARS. Each dot represents each individual observation. The line is the regression line. X axis: correlation of EEG Delta with time. Y axis: change in CARS. **(B)** Change of EEG Theta vs. Change in CARS. Each dot represents each individual observation. The line is the regression line. X axis: correlation of EEG Theta with time. Y axis: change in CARS. **(C)** Delta vs. Theta. X axis: correlation of delta with time. Each dot represents each individual observation. The line is the regression line. Y axis: correlation of theta with time.

The analysis revealed a significant positive correlation between the change in CARS scores and the change in delta waves, indicating that participants who had a reduction in CARS scores also had a reduction in their delta waves (*r* = 0.62, *p* = 0.008, *n* = 17) (see Figure 5A). We also found a significant negative correlation between the power of theta waves and the change in the CARS scores (r = −0.66, *p* = 0.004, *n* = 17) (Figure 5B); this indicates that participants who had a decrease in CARS scores experienced an increase in the power of their theta waves (*n* = 17). These findings suggest a possible relationship between behavioral symptoms (measured by CARS) and the distribution of brainwaves. We also found a negative correlation between the change in the power of delta waves and the change in the power of theta waves (r = −0.52, *p* = 0.03, *n* = 17) (Figure 5C).

In addition, we analyzed the changes in the power of the ratios of brainwaves over the course of the trial. There were no significant changes.

## Discussion

Our study demonstrated that stimulation of targeted brain areas with pulsed NIR light significantly improved ASD symptoms in two- to six-year-old children, as measured by changes in CARS, EEG, and parental interviews. There were no adverse reactions to treatment in the Active group, and foreseeable side effects, e.g., hyperactivity and headaches, were mild and did not require medical attention. The number and intensity of side effects experienced in the Active group did not differ significantly from the number and intensity of side effects reported in the Sham group, attesting both to the safety of treatment and the success of parental blinding.

In evaluating treatment efficacy, a pre- and post-treatment improvement of 4.5 points in the CARS score is identified as the minimum clinically important difference (76). A mean difference of 4.5 points or more between treatments, indicating a statistically significant difference, suggests numerous cases exceed the minimum clinically relevant threshold, even accounting for a possible placebo effect (1 or 2 points) in the control group. Given the sample size under consideration, achieving such a statistically significant difference between groups was anticipated.

Several previous studies have demonstrated that tPBM could be an effective treatment of symptoms of ASD (72, 74, 75, 77, 78). We hypothesized that several areas of the brain are involved in ASD symptomatology, including the: cortical nodes of the default mode network (DMN), which has been linked to social cognition; DMN underconnectivity has been reported in adults and adolescents with ASD (82); the occipital lobe (probably reaching the cerebellum), as many studies have shown that abnormalities in cerebellar structures are linked to deficits in social cognition as well as restrictive and repetitive behaviors (10), and the Broca and Wernicke areas, since they are mostly involved in language reception and production. Future imaging studies (e.g., fMRI) are needed to further explain the effect of treatment on the target areas of the brain itself, as well as on the functional brain connectivity of those areas when stimulated simultaneously (58). Thus, Cognilum design targets the areas affected by ASD.

EEG has long been suggested as an ASD diagnostic method, which contributes in part to identifying the type and severity of the condition (83). However, few studies specifically examined delta waves in the pediatric population of ASD, which challenges the interpretation of our results. For example, excess delta power has been found in individuals with ASD (versus neurotypical individuals) and has been found in both relative and absolute powers (84, 85) and in multiple regions of the brain, including the dorsal midline, parietal, right temporal (84), and frontal cortical areas (86). Delta waves in wakeful states are often associated with various neurological conditions including TBI, chronic hemorrhage, microglial activation, and inflammation (87–89). Furthermore, the presence of delta waves in the wakeful state, measured from intracranial electrodes, was associated with the locations of future seizures and could be used to predict vulnerability to seizures (90). This connection of delta waves and seizures is particularly interesting for people with ASD, as recently many studies have reported differences in EEG delta wave power between individuals with ASD and neurotypical individuals. For example, a large study with 6,000 participants in ASD from a pre-existing research dataset found that about 12% also had epilepsy (91). Additionally, approximately 30% of individuals with ASD had epileptiform EEG, even if they did not experience seizures (92).

The observed abnormal power of delta waves in our participants and its reduction in the Active group over the course of the trial need to be explored in future studies, as it may be potentially a biomarker of ASD and possibly predictive of seizures. In addition, the changes of power of delta were correlated with changes in CARS scores. Therefore, *a reduction in the power of delta waves in the Active group could be a sign of the efficacy of the treatment provided*.

We also observed that the decrease in the CARS scores was significantly correlated with the increase in the power of theta waves. Previous research suggests that theta waves are not sufficiently present in children and adults diagnosed with ASD (93, 94). Therefore, redistribution of delta brain waves from higher to lower power and of theta brain waves from lower to higher power during the series of tPBM treatment might signal a healing or modulating phenomenon occurring as a result of tPBM treatment. These conclusions are highly speculative, and more research is needed to better understand the connection of brain waves (especially delta and theta waves) with ASD, as well as how they could be used to monitor the efficacy of tPBM and their ability to modulate brain physiology.

There are several parameters that determine the exact nature of brain stimulation with tPBM, for example: light wavelength, pulse frequency, power irradiance, frequency of treatment, total duration of treatment, duration of each individual session, and areas of brain stimulation, amongst others. Each of these parameters could potentially be further optimized. For example, each individual session lasted only 6 min and it might be beneficial to increase the duration of each treatment session, especially for older participants or those with a darker skin tone, in which the overall deposition of light energy in the brain is lessened by increased light absorption in dark skin and greater scalp and skull thicknesses. Furthermore, the overall duration of treatment could also be extended beyond 8 weeks. Future dosing studies are necessary to optimize and potentially personalize dosing according to the age of the patients, their skin color, and the type and severity of their symptoms. We believe that tPBM treatment is a brain stimulation technology that can promote functional brain connectivity, which is probably the most effective when used in combination with other methods (including behavioral therapy and increased parental and social interactions).

Sunlight is often considered an alternative to photobiomodulation. Although exposure to sunlight might be beneficial for reducing psychiatric symptoms such as depression, the wavelengths of light in sunlight are different from those used in this trial (e.g., sunlight mostly has waves in the ultraviolet range, and prolonged exposure to UV is cancerogenic and may lead to skin burns). Other parameters of the treatment, such as pulsing and precise power (to establish penetration through skull) are also impossible to replicate with sunlight alone.

## Limitations

It is difficult to draw conclusions from the small sample size of this study because there are many variables that could have contributed to the results. Potentially confounding variables include the increased socialization (e.g., exposure to books, puzzles, educational toys, and musical YouTube videos) of study participants; however, it is unlikely that this exposure alone could explain our results. A large proportion of study participants had already been receiving intense therapies (e.g., they receive one-on-one speech and occupational therapy several times a week, as well as one-on-one ABA therapy every weekday, up to 4 h a day, for a total of 20 h of therapy per week). In addition, most of the participants attended special educational settings, where they also received group therapies and interacted with other children. However, these confounding variables, were controlled by the Sham group.

The long-term individual results of this therapy are likely to vary. First, genetics could be a factor, as our participants did not have genetic tests, and it is possible that for some an ASD diagnosis could be secondary to an undecided genetic condition, such as Fragile X syndrome. Second, the social environment of the individual patient may play a role. Although most of our participants were receiving intensive therapy, some were only scheduled to begin therapy. Unfortunately, some of the participants were overexposed to electronic devices and underexposed to interactions with other children and adults. Although we did not track these conditions systematically, some parents reported an overreliance on electronic devices. Finally, continuous exposure to unknown pathogens that lead to an inflammatory response could be another factor. Our participants were recruited from various environments, and some represented underprivileged groups of population with various living conditions. Future research is necessary to further examine the relative contribution of these variables to the ability of children to respond to various therapeutic interventions, including tPBM, and their overall development trajectory.

It is also possible that parents of participants in the Active group noticed an initial improvement in children and became excited to be able to increase their interactions with children, which could have positively impacted the effect of treatment. Future studies are necessary to measure this effect of increased parental contributions in their interactions with children who start to show improvement in their symptoms.

We recognize that the results of a study with a small sample size of only 30 participants could have been affected by withdrawal and replacement. Future studies with a large sample are necessary to address the issues of participant withdrawal, and to control for potential effects based on gender, functional impairment, verbal status, skin color, and concomitant therapy. Another limitation of this small trial was the heterogeneous sample; ASD has a high comorbidity with other neurological and psychiatric disorders, such as ADHD, OCD, anxiety, and cognitive disabilities. Future research with a large sample size is needed to measure the contribution of all of these variables to the outcome of tPBM treatment. In addition, a large sample size study will allow the examination of each subscale of CARS separately. Future studies will also allow to investigate drug interactions, as many patients with ASD take psychotropic medications, such as risperidone and methylphenidate, on a regular basis.

In addition, it should be noted that time scaling was performed for EEG analysis, to ensure that data from all participants had equal weight in the analysis, to adjust for variability in the total treatment duration and the timing of EEG observations for each participant. Future studies with larger sample sizes are needed to reduce the variability and the need for such scaling.

Future research is needed to clarify the optimal doses of tPBM, particularly for nonwhite patients who may require higher treatment doses due to greater light absorption losses by higher levels of skin melanin, which reduces the penetration of light into brain tissues. More research is also needed to determine the long-term efficacy and possible side effects of the treatment.

## Conclusion

This randomized, sham controlled study showed that stimulation of selected/targeted areas of the brain with 850 nm NIR light reduced ASD symptoms, measured by CARS scores, and affects brain electrophysiology (EEG oscillations). Therefore, tPBM using Cognilum™ could be a safe and effective therapy to reduce the symptoms of ASD. Additional studies are needed to replicate and extend the reported effects, as well as establish the most effective personalized dose based on age, sex, skin color, severity, and nature of symptoms, and other factors. Furthermore, the distribution of brain waves should be further investigated as a possible biomarker of ASD. The integration of behavioral data with EEG could be a promising method to understand various subtypes of ASD and evaluate possible treatments.

## Data availability statement

The raw data supporting the conclusions of this article will be made available by the authors, without undue reservation.

## Ethics statement

The studies involving humans were approved by WCG Clinical Services North America. The studies were conducted in accordance with the local legislation and institutional requirements. Written informed consent for participation in this study was provided by the participants' legal guardians/next of kin.

## Author contributions

YF, ES, MN, and AS contributed to conception and design of the study. YF and ES organized the database. YF, ES, and WS performed the statistical analysis. ES and YF wrote the first draft of the manuscript. LT and MN wrote sections of the manuscript. All authors contributed to the article and approved the submitted version.

## Footnotes

^1^https://scipy.org/

^2^https://www.statsmodels.org/

^3^https://matplotlib.org/

^4^www.trialfacts.com
